# Distinct competitive and allosteric binding modes of nanobodies targeting EphA4

**DOI:** 10.1016/j.jbc.2026.113394

**Published:** 2026-08-05

**Authors:** He Jiang, Lulu Ding, Chenlu Yang, Guankai Guo, Chao Zhong, Zhiqiang Ma, Haoran Zhu, Jiahua Zhang, Yang Luo, Zhen Liu, Rui Liu, Yu Ding

**Affiliations:** 1 State Key Laboratory of Genetics and Development of Complex Phenotypes, School of Life Sciences, Fudan University, Shanghai, China; 2 Quzhou Fudan Institute, Quzhou, Zhejiang, China

**Keywords:** EphA4, antibody, nanobody, ALS, structure

## Abstract

Erythropoietin-producing hepatocellular carcinoma A4 (EphA4) signaling is a key negative regulator of axonal regeneration and a genetic modifier of ALS, making EphA4 an attractive but mechanistically underexplored therapeutic target. Although EphA4-targeting nanobodies have shown inhibitory potential, the structural principles governing their binding and inhibitory mechanisms remain largely unknown. Here, we report high-resolution crystal structures of the EphA4 ligand-binding domain in complex with four nanobodies (Nb50, Nb53, Nb57, and Nb60), resolved at 1.30 to 2.21 Å. Nb50, Nb53, and Nb57 competitively engage the canonical ephrin-binding pocket through deep complementarity-determining region 3 insertion, directly mimicking ephrin recognition. In contrast, Nb60 adopts a previously unrecognized binding mode, contacting two EphA4 molecules at noncanonical sites in the crystal structure through framework-dominated interactions and supporting a structural model for steric and allosteric restriction of ephrin access. Integrated biophysical analyses reveal distinct thermodynamic and kinetic signatures underlying these binding mechanisms. Together, our findings uncover unexpected structural diversity in nanobody-mediated EphA4 recognition and provide a framework for rational development of EphA4-targeted modulators for ALS and related neurodegenerative conditions.

ALS is a fatal neurodegenerative disorder classified as a motor neuron disease, characterized by the progressive degeneration of upper and lower motor neurons, leading to muscle weakness, atrophy, spasticity, and ultimately respiratory failure (1, 2, 3). Most patients die within 3 to 5 years after disease onset, and the incidence of ALS continues to increase worldwide, reflecting its complex and multifactorial etiology (4, 5, 6). Although disease-modifying agents such as riluzole, edaravone, and sodium phenylbutyrate/taurursodiol modestly slow disease progression, their clinical efficacy remains limited (7, 8, 9, 10). Despite strong genetic and functional evidence implicating erythropoietin-producing hepatocellular carcinoma A4 (EphA4) in ALS progression, how EphA4 can be selectively and mechanistically inhibited at the extracellular level remains unclear.

EphA4 is a receptor tyrosine kinase that plays critical roles in nervous system development, synaptic plasticity, and neural injury responses (11, 12, 13, 14, 15, 16, 17, 18, 19). Increasing evidence has linked EphA4 signaling to motor neuron degeneration in ALS (20, 21, 22, 23). Inhibition of EphA4 signaling rescues ALS-associated phenotypes in zebrafish models carrying SOD1 or TDP-43 mutations (24, 25), while EphA4-deficient mice exhibit enhanced axonal regeneration and functional recovery following spinal cord injury (26, 27, 28, 29, 30). Mechanistically, ephrin-A1-mediated activation of EphA4 triggers the Ephexin1-RhoA-ROCK-myosin II cascade, which suppresses axonal regrowth after neural injury (30, 31, 32, 33, 34). Together, these findings establish EphA4 as a compelling therapeutic target for ALS intervention (35, 36).

As a single-pass transmembrane receptor, EphA4 contains an extracellular ligand-binding domain (LBD) that exhibits substantial sequence and conformational divergence from other Eph family members and is spatially separated from the conserved intracellular ATP-binding kinase domain, rendering it an attractive target for selective inhibition (37, 38, 39). Nanobodies (Nbs), characterized by their small size, high stability, strong affinity, and low immunogenicity, have emerged as versatile tools for molecular recognition and therapeutic targeting, with increasing applications in clinical diagnostics and targeted therapy (40, 41, 42, 43, 44, 45, 46, 47, 48, 49). Although several EphA4-targeting Nbs have been identified (50), it remains unknown whether they act through direct competition with ephrin ligands or through alternative, allosteric mechanisms, and no structural information is currently available to resolve these questions.

To address this limitation, we determined high-resolution crystal structures of the EphA4 LBD bound to multiple Nbs and combined these data with systematic biochemical and biophysical characterization. This integrated analysis uncovers two fundamentally distinct EphA4-targeting strategies: canonical competitive engagement of the ephrin-binding pocket and a previously unrecognized noncanonical binding mode mediated by Nb60. Unlike other Nbs, Nb60 engages a unique epitope on the EphA4 LBD and exhibits a broader inhibitory binding mode. Together, these findings provide a structural and biochemical framework for understanding Nb-mediated EphA4 recognition and modulation and inform the rational design of EphA4-targeted therapeutic strategies for ALS.

## Results

### MALDI-TOF validation of EphA4 and Nb expression and complex formation

**Figure 1:**
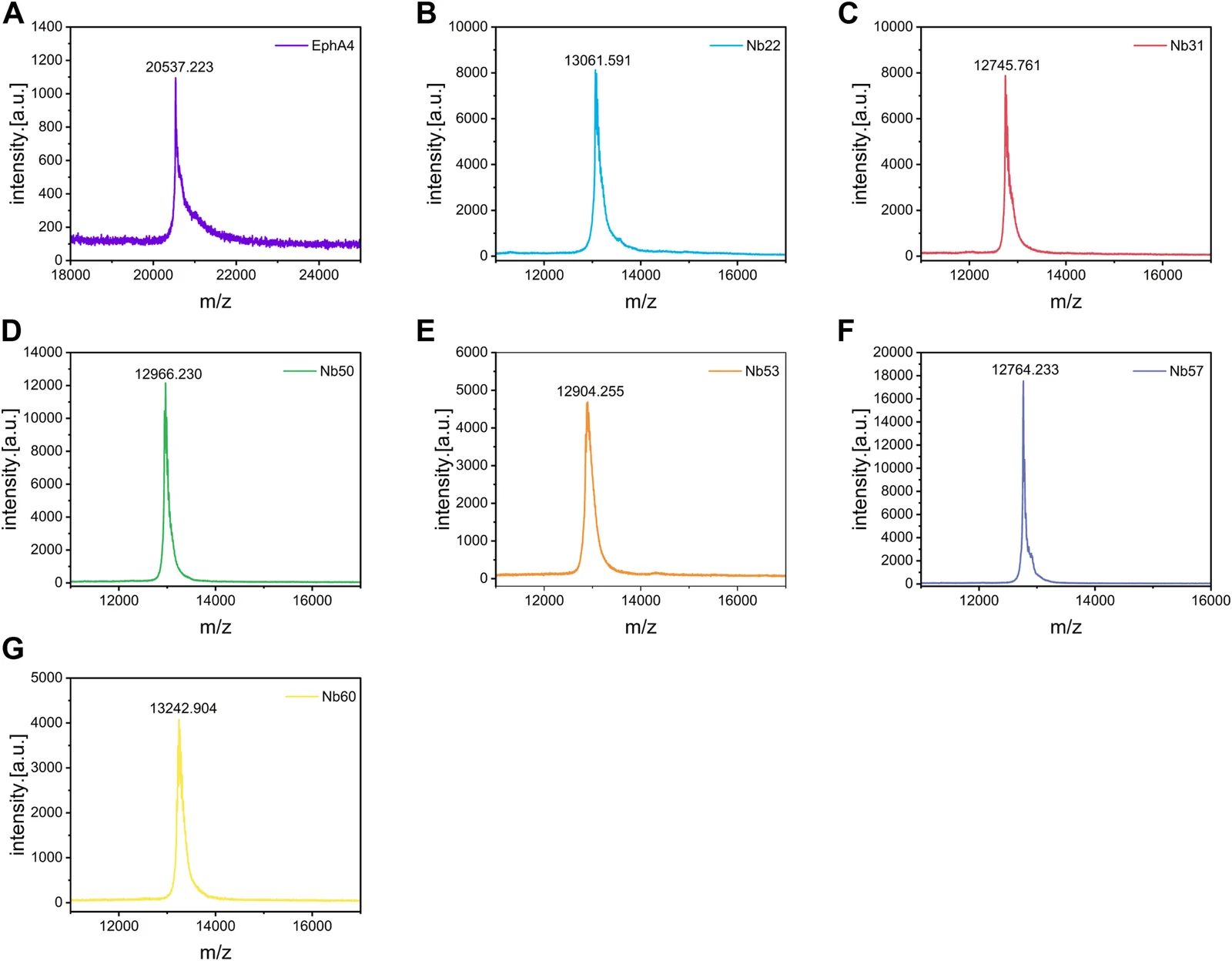
**MALDI-TOF analysis of EphA4 LBD and nanobodies.** Recombinant proteins were expressed and purified to homogeneity, followed by buffer exchange into ammonium acetate prior to analysis. Molecular mass determination was performed using linear-mode MALDI-TOF mass spectrometry. The horizontal axis represents mass-to-charge ratio (m/z), and the vertical axis represents intensity, with peak positions indicating the corresponding m/z values. *A*, EphA4 LBD. *B*, Nb22. *C*, Nb31. *D*, Nb50. *E*, Nb53. *F*, Nb57. *G*, Nb60. LBD, ligand-binding domain; Nb, nanobody.

Six Nbs previously reported were selected for analysis. Nb22, Nb31, Nb53, and Nb57 exhibited high specificity toward EphA4, whereas Nb50 and Nb60 showed broader binding profiles within the EphA family; Nb60 also displayed appreciable affinity for EphB2. The EphA4 LBD and six Nbs were recombinantly expressed in *Escherichia coli* and purified to apparent homogeneity (>95% purity) by affinity chromatography (protein sequences are provided in Table S1), followed by ion-exchange and size-exclusion chromatography. MALDI-TOF MS confirmed molecular masses of approximately 13 kDa for all Nbs and ∼20 kDa for the EphA4 LBD, consistent with theoretical values (Fig. 1). To evaluate complex formation, EphA4 LBD was incubated with excess Nb and subjected to size-exclusion chromatography, yielding six well-resolved EphA4-Nb complexes (Figs. S1–S3).

### Structural and thermal stability of EphA4 and Nb complexes

**Figure 2:**
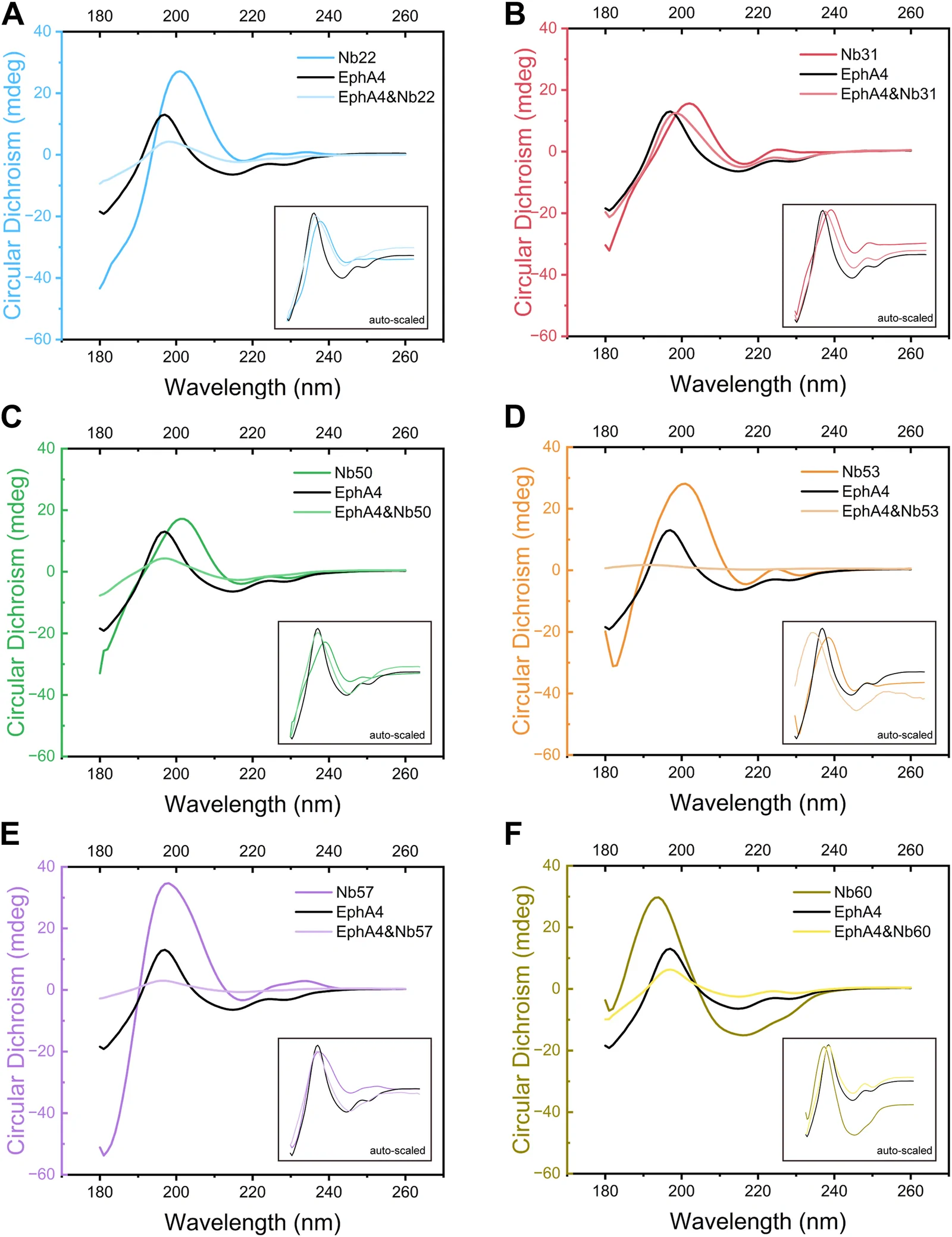
**Circular dichroism analysis of EphA4 LBD, nanobodies (Nbs), and their complexes.** Far-UV CD spectra of EphA4 LBD, individual nanobodies, and the corresponding EphA4-nanobody complexes are shown. The main panels are plotted using the same x-axis and y-axis ranges to facilitate direct comparison among samples. Insets show autoscaled views of the same spectra to better visualize low-amplitude traces. The horizontal axis denotes wavelength (nm), and the vertical axis represents ellipticity (mdeg). *A*, EphA4, Nb22, and EphA4-Nb22. *B*, EphA4, Nb31, and EphA4-Nb31. *C*, EphA4, Nb50, and EphA4-Nb50. *D*, EphA4, Nb53, and EphA4-Nb53. *E,* EphA4, Nb57, and EphA4-Nb57. *F*, EphA4, Nb60, and EphA4-Nb60. EphA, erythropoietin-producing hepatocellular carcinoma A; LBD, ligand-binding domain.

Prior to crystallographic studies, the secondary structure composition of EphA4 and the Nbs was assessed by CD spectroscopy (Fig. 2). The EphA4 LBD displayed a β-sheet-rich architecture dominated by antiparallel β-strands, consistent with its reported β-sandwich fold of EphA4 (51, 52) (Figs. S4 and S5). Nb22, Nb31, Nb50, and Nb53 exhibited typical Nb secondary structure profiles characterized by high antiparallel β-sheet content and minimal α-helical contribution. In contrast, Nb57 showed a reduced antiparallel β-sheet proportion with a relative increase in parallel β-sheet content, whereas Nb60 displayed a distinct profile with markedly elevated α-helical content, indicating structural variability among the Nbs (Table 1).

**Table 1:** Secondary structure composition of EphA4 LBD, nanobodies, and their complexes determined by CD spectroscopy

Sample ID	Helix	Anti-parallel β-sheet	Parallel β-sheet	β-turn	Random coil
EphA4	8.84%	40.67%	4.13%	19.55%	26.82%
Nb22	7.71%	44.26%	3.66%	16.01%	28.35%
Nb31	5.49%	44.56%	5.10%	14.92%	29.93%
Nb50	5.68%	46.83%	4.92%	14.57%	28.10%
Nb53	8.32%	45.98%	4.21%	15.01%	26.48%
Nb57	6.08%	36.46%	7.62%	18.13%	31.82%
Nb60	13.61%	42.50%	3.26%	17.89%	22.74%
EphA4-Nb22	9.46%	42.88%	3.06%	17.67%	26.93%
EphA4-Nb31	6.74%	43.21%	4.99%	16.72%	28.35%
EphA4-Nb50	8.67%	43.71%	3.52%	17.62%	26.57%
EphA4-Nb53	12.03%	43.99%	2.11%	17.63%	24.24%
EphA4-Nb57	7.50%	45.92%	4.84%	16.70%	24.95%
EphA4-Nb60	8.70%	43.98%	3.63%	18.36%	25.43%

Secondary structure contents were estimated using the CDNN software. To account for the intrinsic ±5% uncertainty of the analysis, the total percentages were proportionally normalized to 100%.

Secondary structure changes upon complex formation were subsequently examined. For all EphA4-nanobody complexes except EphA4-Nb60, complex assembly resulted in increased α-helical content accompanied by reduced random coil contributions, suggesting modest conformational rearrangement upon binding. The EphA4-Nb57 complex exhibited the most pronounced reduction in random coil content together with a substantial increase in antiparallel β-sheet structure, while the EphA4-Nb53 complex showed a notable increase in α-helicity. In contrast, the EphA4-Nb60 complex displayed an opposing trend, with increased disorder and reduced α-helical content, consistent with its distinct binding behavior observed in subsequent structural analyses.

**Figure 3:**
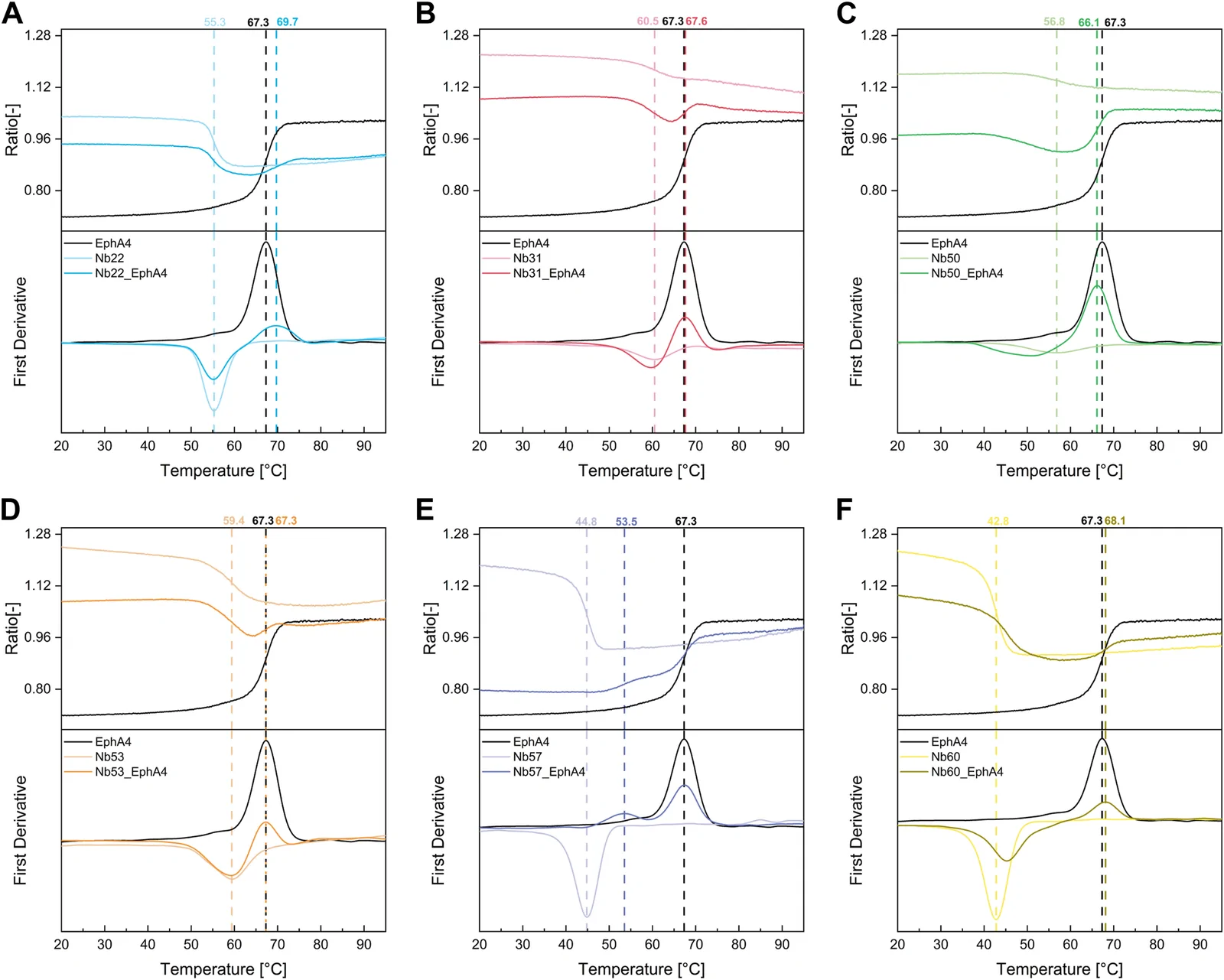
**NanoDSF analysis of EphA4 LBD, nanobodies, and their complexes.** NanoDSF thermal unfolding profiles of individual nanobodies, EphA4 LBD, and the corresponding EphA4-nanobody complexes are shown. The *upper traces* show the fluorescence ratio, and the *lower traces* show the first derivative of the ratio signal. *Vertical dashed lines* indicate only the inflection-point melting temperatures (Tm) of the corresponding samples; onset/Tagg values are not marked in the figure. Values shown above each panel indicate the corresponding Tm values. *A*, EphA4, Nb22, and Nb22_EphA4. *B*, EphA4, Nb31, and Nb31_EphA4. *C*, EphA4, Nb50, and Nb50_EphA4. *D*, EphA4, Nb53, and Nb53_EphA4. *E*, EphA4, Nb57, and Nb57_EphA4. *F*, EphA4, Nb60, and Nb60_EphA4. NanoDSF, nano differential scanning fluorimetry; EphA, erythropoietin-producing hepatocellular carcinoma A; LBD, ligand-binding domain.

Thermal stability was further evaluated using NanoDSF analysis. Because Nbs were added in excess, the NanoDSF unfolding traces of the mixtures typically showed two transitions, consistent with a population of free Nb coexisting with EphA4-bound species. The first transition (Tm1) falls within the melting range of the isolated Nbs, whereas the second transition aligns with the unfolding temperature of EphA4 LBD (Tm = 67.3 °C). We therefore used the second transition as an apparent melting temperature reporting the stability of EphA4-Nb complexes. Consistent with complex formation, the Nb-associated transition (Tm1) shifted to higher temperatures compared with Nb monomers, indicating increased nanobody thermal stability upon EphA4 binding. Notably, Tm2 values (66.1–69.7 °C) were close to that of EphA4 alone, suggesting that complex formation does not markedly perturb the global thermal stability of the LBD under these conditions (Fig. 3 and Table 2).

**Table 2:** Thermal stability parameters of EphA4 LBD, nanobodies, and their complexes determined by NanoDSF

Sample ID	Onset points (Tagg)	Inflection point 1 (Tm1)	Inflection point 2 (Tm2)
EphA4	61.1 °C	67.3 °C	
Nb22	51.8 °C	55.3 °C	
Nb31	52.2 °C	60.5 °C	
Nb50	49.1 °C	56.8 °C	
Nb53	51.1 °C	59.4 °C	
Nb57	38.6 °C	44.8 °C	
Nb60	36.1 °C	42.8 °C	
Nb22 with EphA4	51.6 °C	55.2 °C	69.7 °C
Nb31 with EphA4	51.5 °C	59.5 °C	67.6 °C
Nb50 with EphA4	40.6 °C	50.7 °C	66.1 °C
Nb53 with EphA4	50.2 °C	59.0 °C	67.3 °C
Nb57 with EphA4	48.8 °C	53.5 °C	67.4 °C
Nb60 with EphA4	35.2 °C	45.2 °C	68.1 °C

Onset points indicate the temperatures at which protein aggregation begins, whereas inflection points correspond to the temperatures at which 50% of the protein population is thermally unfolded (Tm).

### The crystal structure of Nbs targeting distinct epitopes of EphA4 LBD

To elucidate the structural mechanisms by which different Nbs modulate EphA4, crystal structures of the EphA4 LBD in complex with Nb50, Nb53, Nb57, and Nb60 were determined using X-ray crystallography. In these structures, EphA4 adopts a 1:1 binding stoichiometry with Nb50, Nb53, and Nb57, whereas a distinct 4:2 binding mode was observed for the EphA4-Nb60 complex. Detailed data collection and refinement statistics are summarized in Table 3.

**Table 3:** Data collection and refinement statistics for EphA4-nanobody complexes

Diffraction source	EphA4_Nb50	EphA4_Nb53	EphA4_Nb57	EphA4_Nb60
PDB entry	23UD	23UE	23UF	23UG
Space group	P 2_1_ 2_1_ 2	I 1 2 1	P 6_1_ 2 2	P 1 2_1_ 1
a, b, c (Å)	55.72 122.37 35.40	69.04 43.11 97.11	77.15 77.15 180.91	56.62 147.85 64.67
α, β, γ (◦)	90.0 90.0 90.0	90.0 91.3 90.0	90.0 90.0 120.0	90.0 92.5 90.0
Resolution (Å)	27.86–1.51 (1.57–1.51)	55.66–1.30 (1.35–1.30)	44.77–2.21 (2.29–2.21)	48.65–1.85 (1.92–1.85)
I/σ(I)	8.86 (2.02)	8.88 (3.37)	21.20 (2.62)	11.54 (1.31)
Completeness (%)	99.53 (97.29)	98.39 (96.29)	97.36 (89.83)	99.87 (99.58)
Number of unique reflections	32,283 (3700)	69,303 (6748)	16,280 (1459)	90,194 (8951)
Rwork	0.2054 (0.4054)	0.1746 (0.2225)	0.2570 (0.3451)	0.2036 (0.3502)
Rfree	0.2395 (0.4561)	0.1919 (0.2213)	0.3196 (0.4030)	0.2396 (0.3484)
RMSD				
Bond length (Å)	0.010	0.006	0.036	0.021
Bond angles (◦)	1.09	0.94	1.46	1.16
Average B, all atoms (Å^2^)	33.87	14.58	42.75	37.40

Crystallographic data and refinement statistics for the EphA4-Nb50, EphA4-Nb53, EphA4-Nb57, and EphA4-Nb60 complexes.

**Figure 4:**
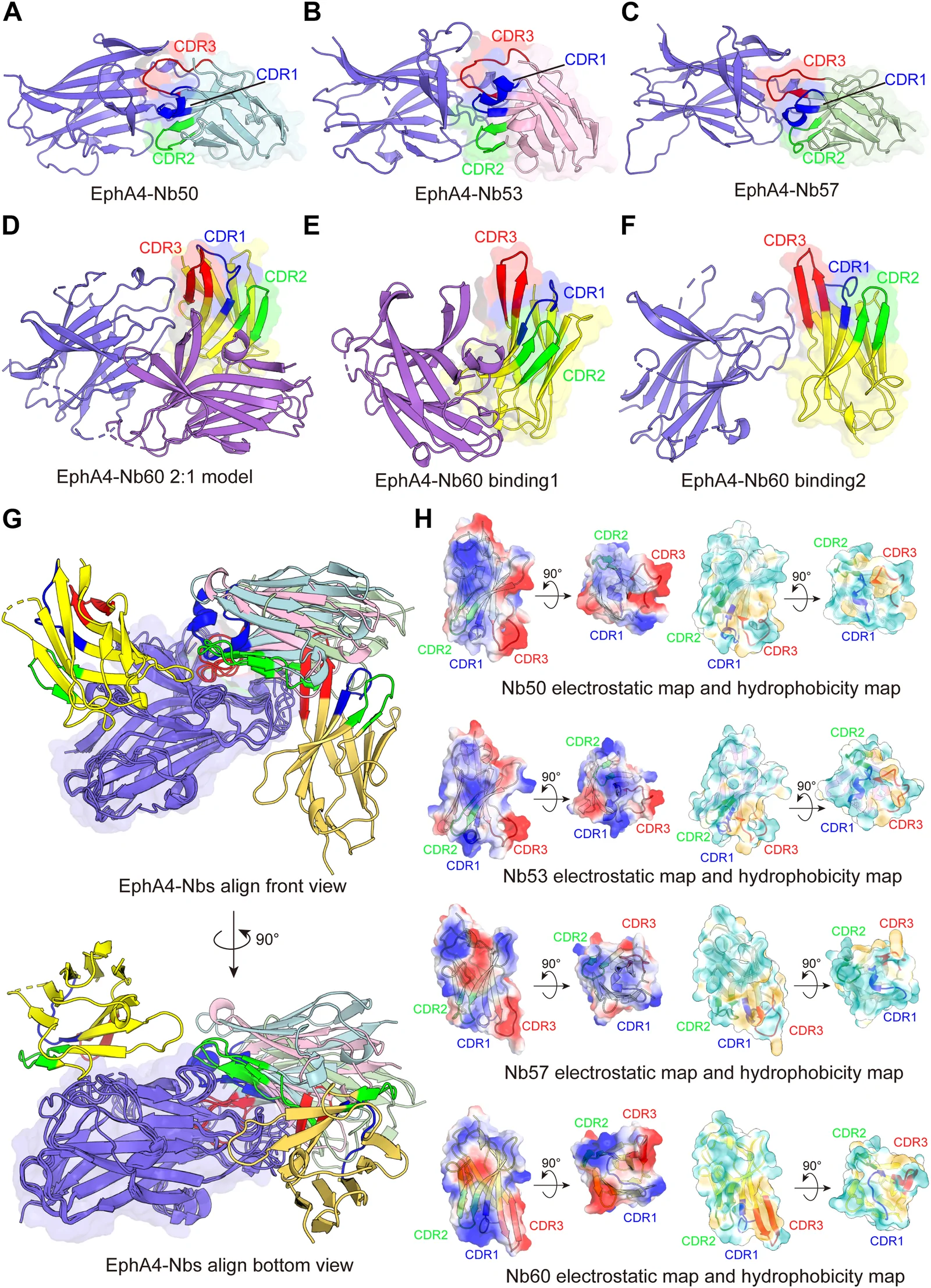
**X-ray crystallographic structures of EphA4 LBD-nanobody (Nb) complexes.***A*, *B*, and *C*, overall structure of EphA4-Nb50 (*A*), EphA4-Nb53 (*B*), and EphA4-Nb57 (*C*). EphA4 LBD is shown in *purple-blue*, Nb50 in *light blue*, Nb53 in *light pink*, Nb57 in *light green*, Complementarity-determining regions (CDRs) are colored as follows: CDR1 (*blue*), CDR2 (*green*), and CDR3 (*red*). *D*, *E*, and *F*, overall structure of EphA4-Nb60, EphA4 binds Nb60 in a 2:1 stoichiometry (*D*). Two EphA4 LBD molecules are shown, one is *blue-purple* and the other is *purple*, Nb60 in *yellow*. Binding mode 1 is shown in (*E*), and binding mode 2 is shown in (*F*). *G*, structural alignment of EphA4-Nb50, EphA4-Nb53, EphA4-Nb57, and EphA4-Nb60 complexes. *H*, surface electrostatic potential maps (*blue*, positively charged; *red*, negatively charged) and surface hydrophobicity distributions (*cyan*, hydrophilic; *yellow*, hydrophobic) of Nb50, Nb53, Nb57, and Nb60, with all three CDR regions indicated. CDR, complementarity-determining region; EphA, erythropoietin-producing hepatocellular carcinoma A; LBD, ligand-binding domain.

Comparative structural analysis revealed that Nb60 engages EphA4 at binding sites distinct from those recognized by Nb50, Nb53, and Nb57 (Fig. 4, *A*–*C, G*). The latter three Nbs recognize highly similar epitopes located within a cavity formed by residues Gly52-Glu62 and Gln156-Thr168 of the EphA4 LBD, and their binding induces conformational changes in the Arg106-Gly116 region. Although only one binding configuration was directly observed in the resolved EphA4:Nb60 (4:2) complex, analysis of symmetry-related molecules revealed an additional Nb60-binding site on EphA4. Among the two binding modes, Nb60 preferentially associates with protruding surface regions of the EphA4 LBD, highlighting a binding mechanism distinct from that of the other Nbs and underscoring the diversity of EphA4-Nb interaction modes (Fig. 4, *D*–*F*).

**Figure 5:**
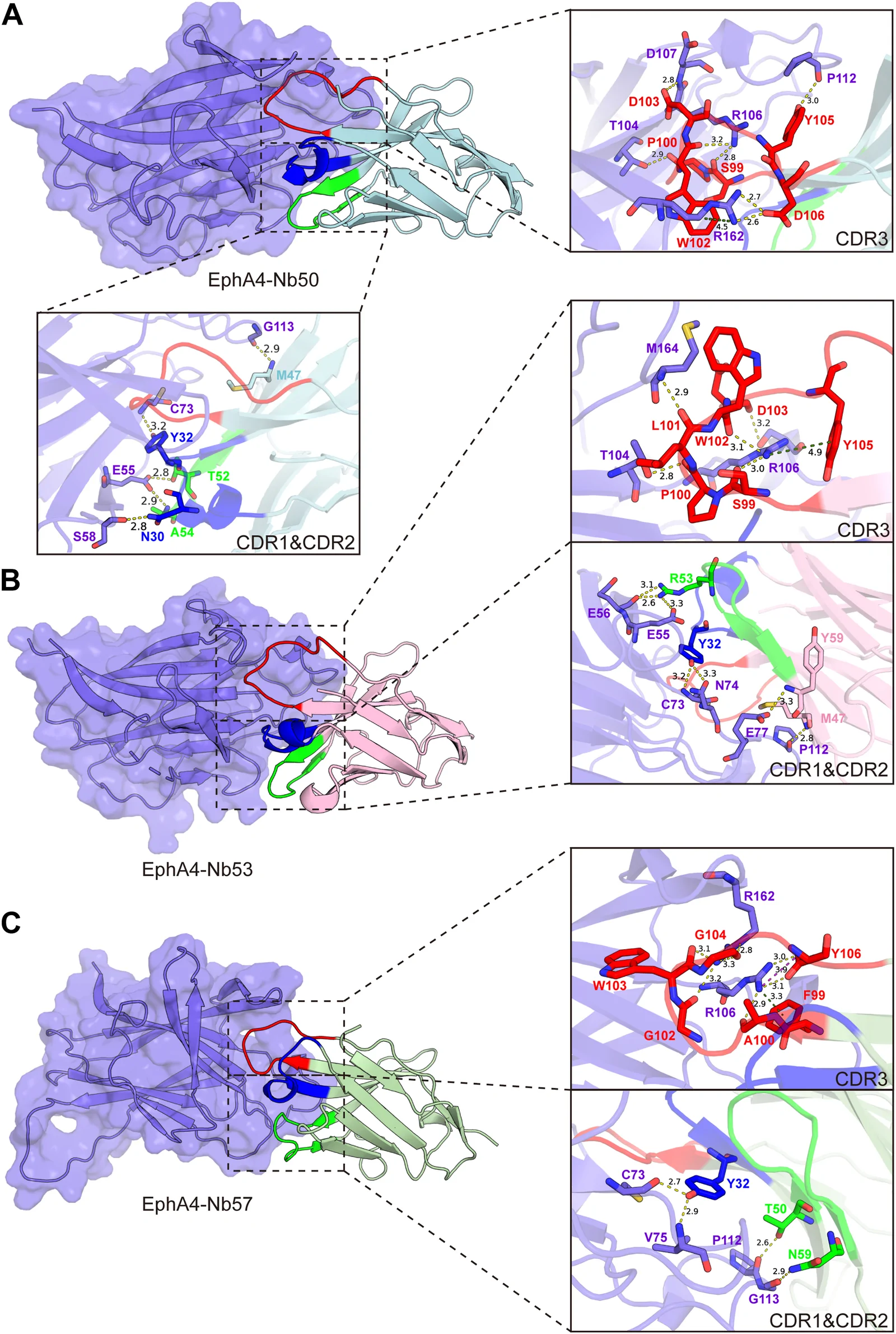
**Detailed binding interfaces of Nb50, Nb53, and Nb57 with EphA4 LBD.***A*, epitope mapping of the EphA4-Nb50 complex. EphA4 LBD is shown in *purple-blue* and Nb50 in *light blue*. CDR1, CDR2, and CDR3 are colored *blue*, *green*, and *red*, respectively. *B*, epitope mapping of the EphA4-Nb53 complex. EphA4 LBD is shown in *purple-blue* and Nb53 in *light pink*. *C*, epitope mapping of the EphA4-Nb57 complex. EphA4 LBD is shown in *purple-blue* and Nb57 in *light green*. CDR, complementarity-determining region; EphA, erythropoietin-producing hepatocellular carcinoma A; LBD, ligand-binding domain; Nb, nanobody.

**Figure 6:**
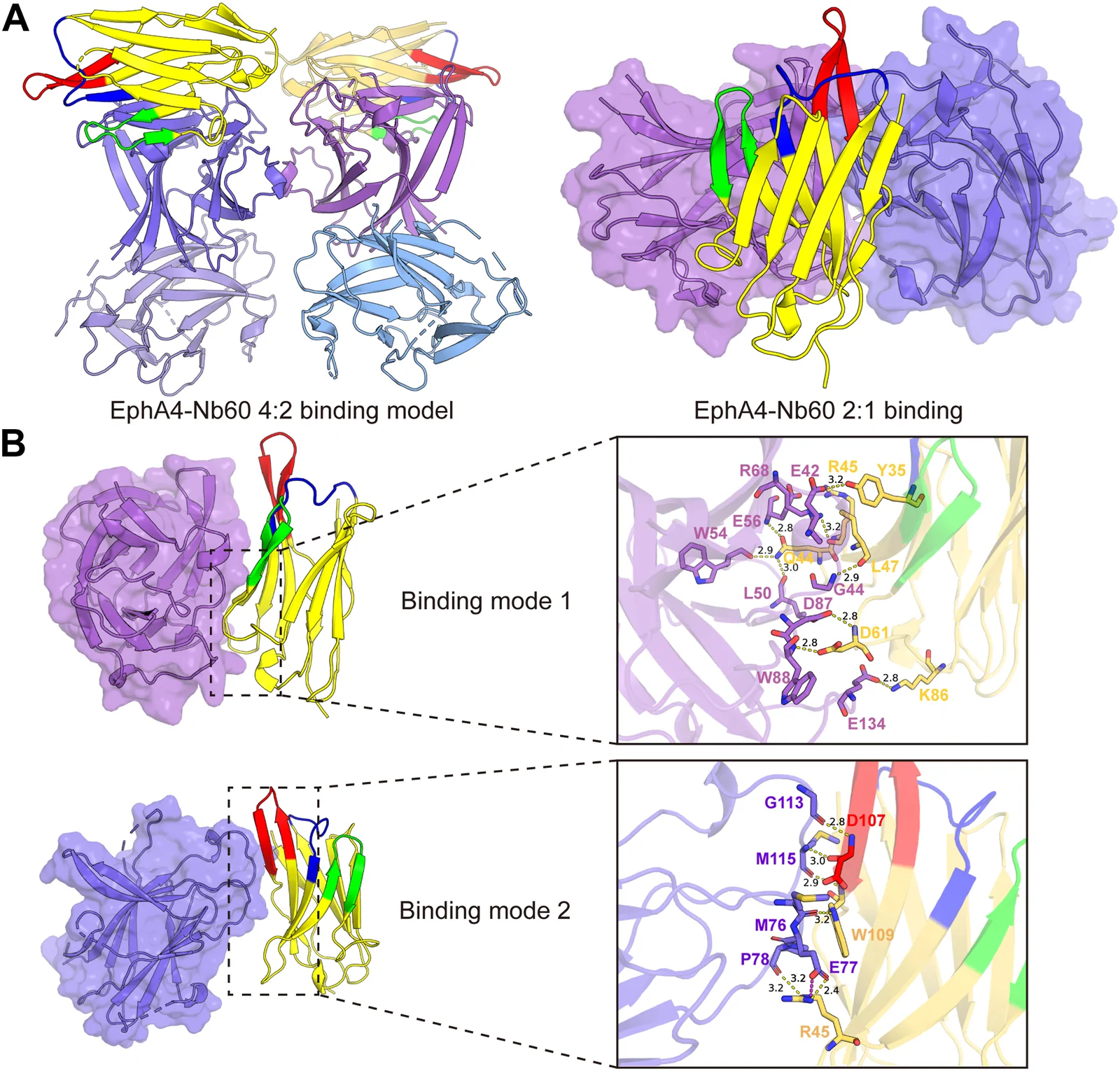
**Detailed binding interfaces and interaction modes of Nb60 with EphA4 LBD.***A*, epitope mapping of the EphA4-Nb60 complex. The resolved crystal structure exhibits an EphA4:Nb60 stoichiometry of 2:1, while a symmetric binding mode corresponding to an EphA4:Nb60 ratio of 2:1 was identified by crystallographic symmetry. EphA4 LBD molecules are shown in different shades of *blue-purple*, and Nb60 is shown in different shades of *orange-yellow*. CDR1, CDR2, and CDR3 are colored *blue*, *green*, and *red*, respectively. *B*, two distinct binding modes of Nb60 interacting with EphA4 LBD. CDR, complementarity-determining region; EphA, erythropoietin-producing hepatocellular carcinoma A; LBD, ligand-binding domain; Nb, nanobody.

Electrostatic surface and hydrophobicity analyses further illustrated distinct complementarity-determining region (CDR) features among the Nbs. Nb50 contains an extended, highly negatively charged CDR3 loop with pronounced hydrophilicity across all three CDRs, suggesting a propensity for forming long-range salt bridges with EphA4. Nb53 displays localized negative charge within its CDR3 loop, which adopts an expanded, cavity-like conformation conducive to close-range interactions, while its CDR1 region exhibits strong positive electrostatic character and CDR3 also includes hydrophobic patches. Nb57 is characterized by a strongly negatively charged CDR3 loop with an elongated hydrophobic tail, suggesting a propensity for forming long-range salt bridges with EphA4. In contrast, Nb60 exhibits relatively weak overall negative electrostatic potential across its CDRs, with a predominantly hydrophobic CDR3 and a strongly positively charged CDR1 region, whereas the framework region between CDR1 and CDR2 displays concentrated negative charge, potentially contributing to its unique binding behavior (Fig. 4*H*).

Structural analysis revealed that Nb50, Nb53, and Nb57 engage EphA4 through a conserved pocket-binding mode. In all three complexes, the elongated CDR3 loop inserts deeply into the concave ligand-binding pocket of the EphA4 LBD, while the shorter CDR1 and CDR2 loops provide additional stabilizing contacts with surrounding surface residues (Fig. 5, *A*–*C*). PDB-PISA analysis indicated comparable interface areas for Nb50 (1072.0 Å^2^), Nb53 (1131.9 Å^2^), and Nb57 (807.6 Å^2^), with favorable binding energies (PISA-predicted Δ^i^G values ranging from −10.9 to −14.4 kcal/mol), supporting the presence of extensive crystallographic interfaces (Table S2) (53).

Across these complexes, EphA4 Arg106 emerges as a central interaction hotspot, mediating extensive hydrogen bonding and π-cation interactions with CDR3 residues, thereby anchoring the Nbs within the binding pocket. Additional contributions from CDR1 and CDR2 residues further reinforce binding through hydrogen bonds and hydrophobic contacts. Notably, Nb53 exhibits a slightly more favorable PISA-predicted interaction energy and broader interface engagement than Nb50, whereas Nb57 displays a more compact binding geometry with a reduced interface area, reflecting subtle differences in interface architecture and spatial complementarity within this shared interaction paradigm.

In contrast to the pocket-binding Nbs, Nb60 engages EphA4 through a fundamentally distinct interaction mode. The EphA4-Nb60 crystal structure reveals a unique 4:2 stoichiometry, arising from the ability of Nb60 to engage EphA4 through two distinct binding configurations observed in the crystal lattice, together with additional EphA4-EphA4 contacts that stabilize the assembly (Fig. 6*A*). Unlike Nb50, Nb53, and Nb57, Nb60 binding is largely independent of its CDR loops and is instead mediated predominantly by framework residues, defining a noncanonical interaction mechanism.

Two distinct Nb60-EphA4 binding configurations were identified. Binding mode 2 is characterized by a small interface area (459.4 Å^2^) and modest PISA-predicted interface Δ^i^G value of −3.1 kcal/mol, involving limited CDR participation and a small number of hydrogen bonds and salt bridges, consistent with a weak or context-dependent secondary contact. Binding mode 1 exhibits a larger interface area (860.0 Å^2^) and a more favorable PISA-predicted interface Δ^i^G value of −6.1 kcal/mol, driven primarily by hydrogen-bonding interactions mediated by framework residues (Fig. 6*B*). Together, these observations suggest that Nb60 can recognize EphA4 through a multivalent, framework-driven binding mode in the crystal structure, which is fundamentally distinct from the CDR3-dominated pocket-binding mode observed for Nb50, Nb53, and Nb57.

### Dynamic light scattering (DLS) analysis of solution-state complex formation

Although high-resolution crystal structures were determined for four EphA4-Nb complexes, all six Nbs formed detectable complexes with EphA4 during solution-state purification and biophysical characterization. To further characterize their solution-state assembly, dynamic light scattering (DLS) was employed to systematically analyze EphA4 LBD, the individual nanobodies, and the corresponding EphA4-Nb complexes.

DLS measurements showed that Nb22, Nb31, Nb50, Nb53, Nb57, and Nb60 exhibited similar hydrodynamic radii of approximately 2.5 nm, whereas monomeric EphA4 LBD displayed a radius of 3.0 nm. Upon complex formation, EphA4-Nb22, EphA4-Nb31, EphA4-Nb50, EphA4-Nb53, and EphA4-Nb57 complexes exhibited increased hydrodynamic radii of 3.6 nm, consistent with formation of compact EphA4-Nb complexes in solution.

**Figure 7:**
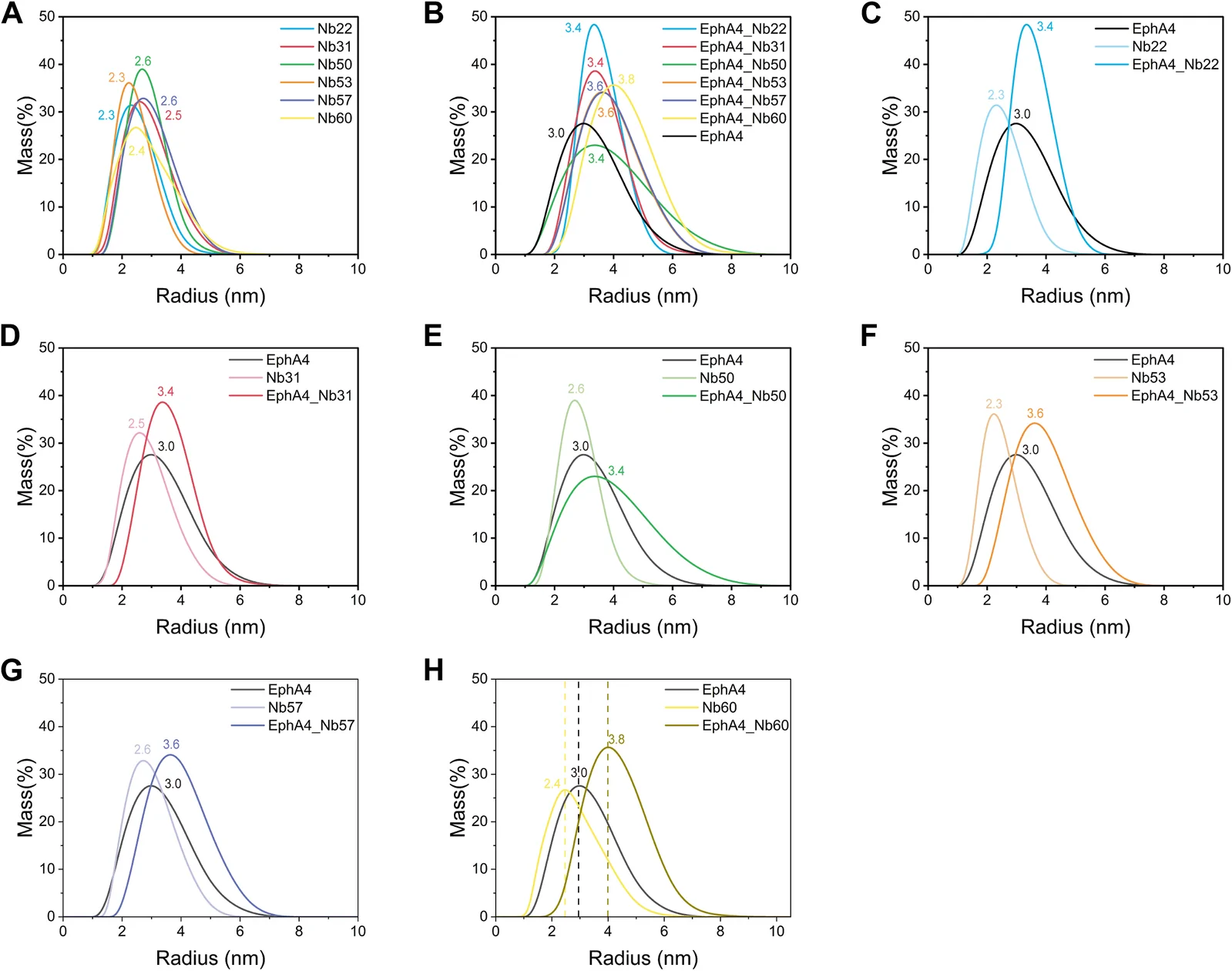
**Dynamic light scattering (DLS) analysis of EphA4 LBD and nanobodies.***A*, DLS-derived hydrodynamic radius distributions of individual Nb22, Nb31, Nb50, Nb53, Nb57, and Nb60. The x-axis denotes particle radius (nm), and the y-axis represents mass percentage (%). Data corresponding to particle radii greater than 10 nm are excluded from display. *B*, DLS-derived hydrodynamic radius distributions of EphA4 LBD alone and in complex with Nb22, Nb31, Nb50, Nb53, Nb57, and Nb60. *C*, *D*, *E*, *F*, *G*, and *H*, DLS-derived hydrodynamic radius distributions of EphA4 LBD, individual nanobodies, and the corresponding EphA4-nanobody complexes. Color coding: Nb22 (*blue*), Nb31 (*red*), Nb50 (*green*), Nb53 (*orange*), Nb57 (*purple*), and Nb60 (*yellow*). EphA, erythropoietin-producing hepatocellular carcinoma A; LBD, ligand-binding domain; Nb, nanobody.

Formation of the EphA4-Nb60 complex increased the hydrodynamic radius to 3.8 nm. Although this value was slightly larger than those of the other EphA4-Nb complexes, the DLS profile showed no evidence of stable dimeric or higher-order EphA4-Nb60 species under the tested conditions (Fig. 7).

### Isothermal titration calorimetry (ITC) analysis of EphA4 LBD-Nb interactions

**Figure 8:**
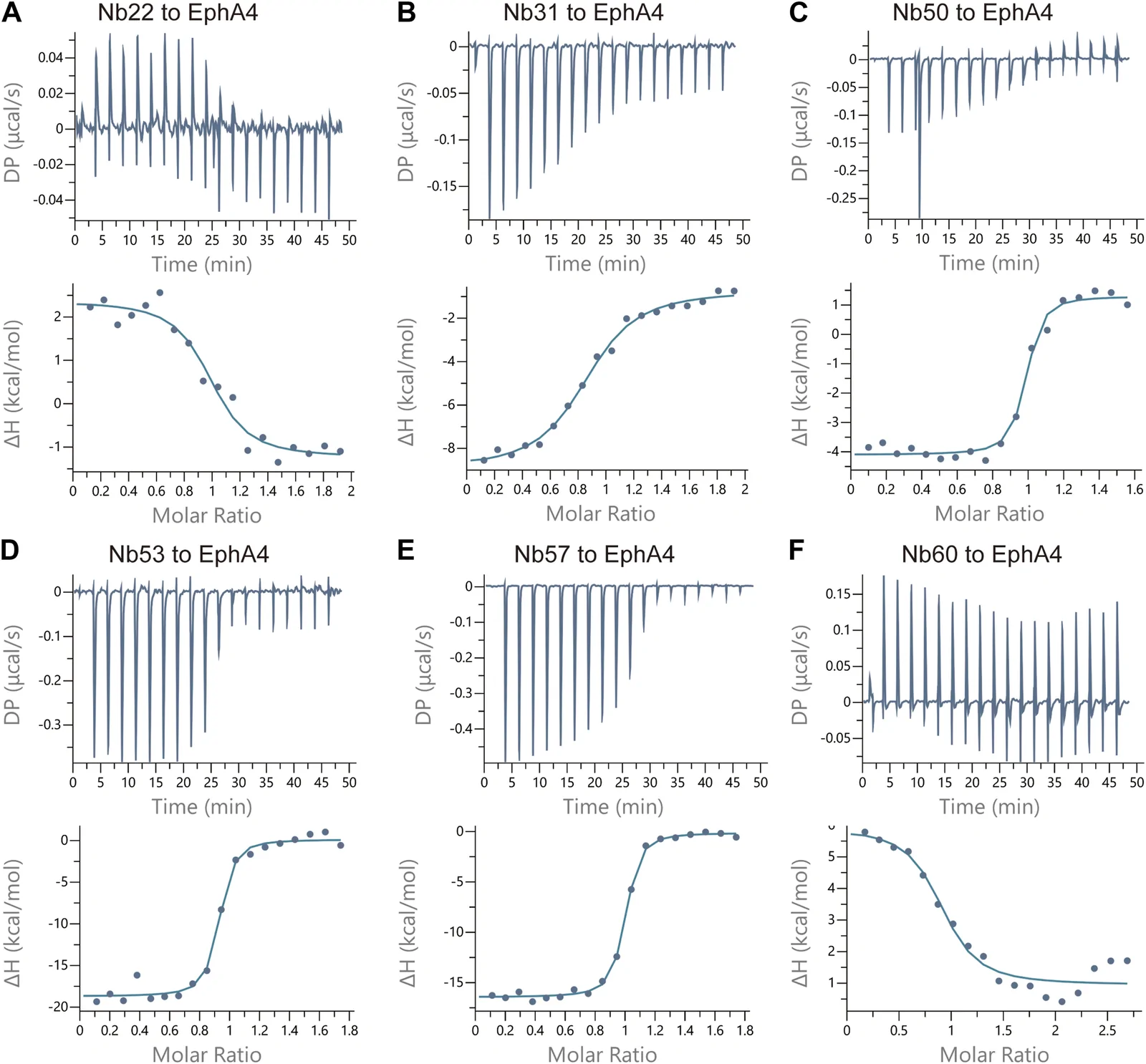
**Isothermal titration calorimetry (ITC) analysis of EphA4 LBD-nanobody (Nb) interactions.** Validation of interactions between EphA4 LBD and nanobodies. The figure shows ITC binding isotherms and the corresponding thermodynamic parameters. *A*, binding of Nb22 to EphA4. *B*, binding of Nb31 to EphA4. *C*, binding of Nb50 to EphA4. *D*, binding of Nb53 to EphA4. *E*, binding of Nb57 to EphA4. *F*, binding of Nb60 to EphA4. EphA, erythropoietin-producing hepatocellular carcinoma A; LBD, ligand-binding domain.

Isothermal titration calorimetry (ITC) was used to quantitatively characterize the interactions between EphA4 LBD and the six Nbs (Fig. 8 and Table 4). The dissociation constants (K_D_) for Nb22, Nb31, Nb50, Nb53, Nb57, and Nb60 were determined to be 47.9 nM, 576 nM, 33.2 nM, 35.6 nM, 31.2 nM, and 358 nM, respectively, indicating relatively higher apparent binding affinities for Nb22, Nb50, Nb53, and Nb57.

**Table 4:** ITC-derived binding affinities of EphA4 LBD with nanobodies

Sample ID	N	ΔH (kcal/mol)	-TΔS (kcal/mol)	ΔG (kcal/mol)	K_D_ (nM)
Nb22 to EphA4	0.676 ± 2.0 × 10^−2^	3.36 ± 0.239	−13.3	−9.94	47.9 ± 43.3
Nb31 to EphA4	0.849 ± 1.8 × 10^−2^	−8.23 ± 0.337	−0.28	−8.51	576 ± 121
Nb50 to EphA4	0.945 ± 9.7 × 10^−3^	−5.39 ± 0.179	−4.81	−10.2	33.2 ± 13.5
Nb53 to EphA4	0.884 ± 9.4 × 10^−3^	−18.8 ± 0.550	8.65	−10.2	35.6 ± 14.0
Nb57 to EphA4	0.954 ± 3.8 × 10^−3^	−16.3 ± 0.188	6.06	−10.2	31.2 ± 4.73
Nb60 to EphA4	0.868 ± 4.9 × 10^−2^	4.98 ± 0.499	−13.8	−8.82	358 ± 218

Summary of dissociation constants (KD) determined by ITC for interactions between EphA4 LBD and the indicated nanobodies.

Analysis of thermodynamic parameters revealed distinct binding driving forces among the Nbs. Nb31, Nb50, Nb53, and Nb57 displayed negative enthalpy changes (ΔH < 0), indicating predominantly enthalpy-driven interactions, likely reflecting polar, electrostatic, and hydrogen-bonding interactions at the binding interfaces.

Among these, Nb31 and Nb50 showed both favorable enthalpic and entropic contributions, although the favorable entropy term was much more pronounced for Nb50 than for Nb31. This mixed enthalpy-entropy contribution is consistent with their productive EphA4 engagement, while the less favorable overall ΔG of Nb31 is consistent with its weaker apparent affinity. In contrast, Nb53 and Nb57 showed strongly favorable enthalpy changes accompanied by unfavorable entropy contributions (-TΔS > 0), suggesting that their binding is predominantly enthalpy-driven and associated with increased conformational ordering upon complex formation. Nb22 and Nb60 displayed positive enthalpy changes but strongly favorable entropy contributions (-TΔS < 0), indicating that their binding is more strongly entropy-driven, potentially due to solvent release or reduced conformational restriction.

### Structure-guided engineering of Nb60 improves EphA4-over-EphA6 selectivity

To further evaluate whether the noncanonical Nb60 interface could be rationally tuned to improve EphA4 specificity, we performed biolayer interferometry (BLI) based binding analyses. As a baseline, all six Nbs showed measurable binding to EphA4 LBD, with apparent affinities broadly consistent with the ITC measurements and previously reported EphA4-binding profiles (Fig. S6, Table S3). We then focused on Nb60 because it was previously reported to display broader Eph receptor cross-reactivity (50). EphA6 was selected as a representative closely related receptor for specificity comparison.

**Figure 9:**
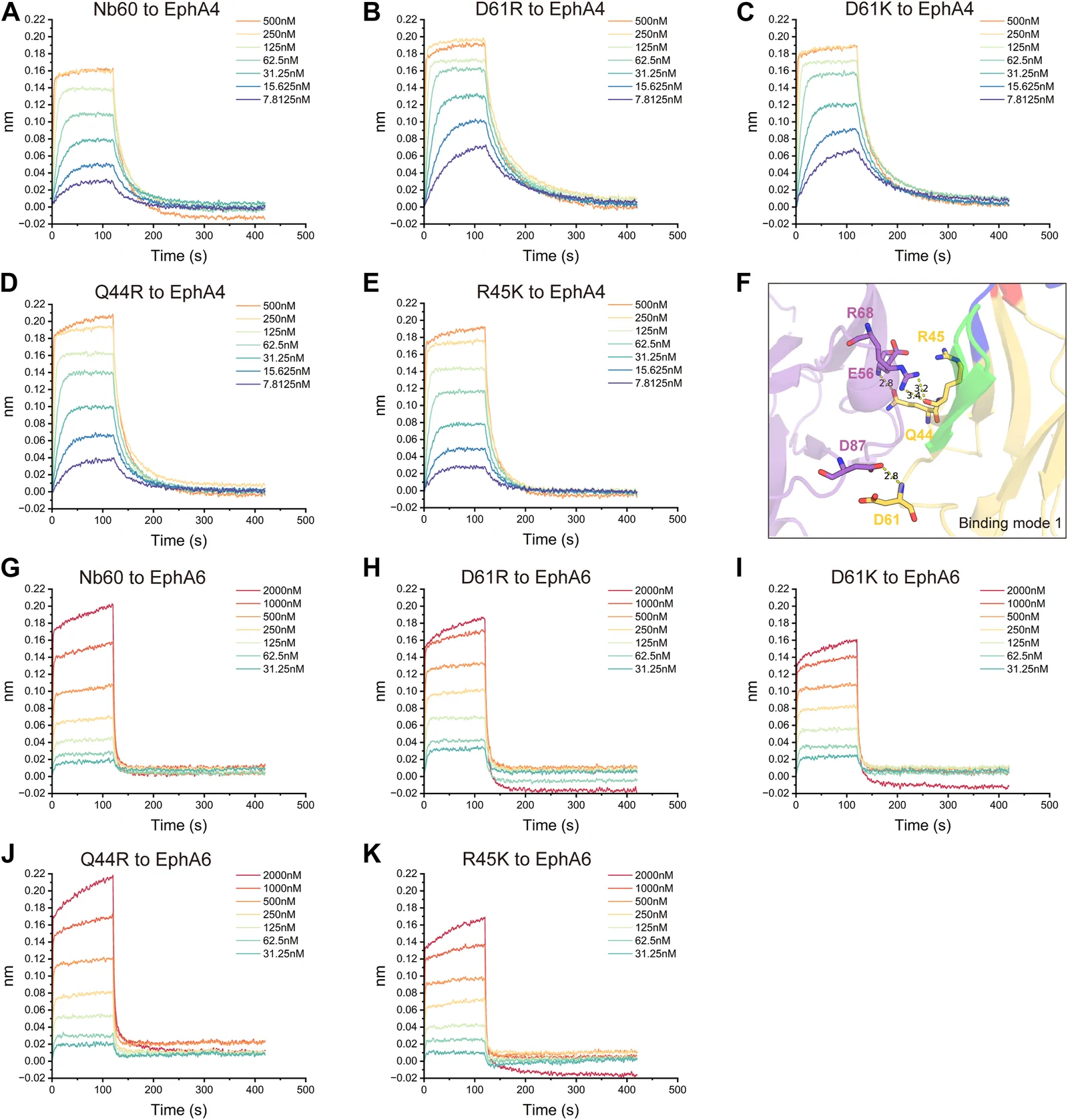
**Structure-guided mutagenesis of Nb60 and BLI analysis of EphA4/EphA6 binding.***A*, *B*, *C*, *D*, and *E*, representative BLI sensorgrams showing binding of wild-type Nb60 and the indicated Nb60 variants to immobilized EphA4 LBD. His-tagged EphA4 LBD was loaded onto NTA biosensors, and Nb60, D61R, D61K, Q44R, or R45K was tested as the soluble analyte using the indicated concentration series. *F*, structural mapping of Nb60 residues selected for mutagenesis at the EphA4-Nb60 interface. EphA4 LBD is shown in *purple*, and Nb60 is shown in *yellow*, with residues Q44, R45, and D61 highlighted. *G*, *H*, *I*, *J*, and *K*, representative BLI sensorgrams showing binding of wild-type Nb60 and the indicated Nb60 variants to immobilized EphA6 LBD under the same assay format. Nb, nanobody; BLI, biolayer interferometry; EphA, erythropoietin-producing hepatocellular carcinoma A; LBD, ligand-binding domain.

To guide mutagenesis, we compared the EphA4 and EphA6 sequences in regions corresponding to the Nb60-contacting surface and mapped divergent receptor residues onto the EphA4-Nb60 structure (Fig. S5). Nb60 residues positioned near these receptor-divergent regions, including D61, Q44, and R45, were selected for mutagenesis, yielding four variants: D61R, D61K, Q44R, and R45K (Fig. 9*F*). Binding of WT Nb60 and the four variants to EphA4 and EphA6 was then quantified by BLI.

Consistent with the previously reported cross-reactivity of Nb60, WT Nb60 showed measurable binding to both EphA4 and EphA6 in our BLI assay. Under this immobilized-receptor assay format, Nb60 bound EphA4 with an apparent KD of 46.7 nM and EphA6 with an apparent KD of 829 nM, corresponding to an approximately 17.8-fold apparent EphA4-over-EphA6 preference. Notably, D61R and D61K improved apparent binding to EphA4, with KD values of 14.8 nM and 16.0 nM, respectively, while maintaining weaker apparent binding to EphA6. As a result, the apparent EphA4-over-EphA6 selectivity increased to approximately 33.1-fold for D61R and 30.4-fold for D61K under the same BLI assay format. In contrast, Q44R improved apparent binding to both EphA4 and EphA6 and therefore did not enhance selectivity, whereas R45K produced a more modest selectivity shift mainly by weakening binding to EphA6 (Fig. 9, *A*–*E*, *G*–*K*, Table 5).

**Table 5:** BLI-derived kinetic parameters for interactions between Eph receptor LBDs and Nb60 variants

Sample ID	k_a_ (1/Ms)	k_dis_ (1/s)	K_D_ (M)
Nb60 to EphA4	8.40 × 10^5^	3.92 × 10^−2^	4.67 × 10^−8^
D61R to EphA4	1.33 × 10^6^	1.96 × 10^−2^	1.48 × 10^−8^
D61K to EphA4	1.33 × 10^6^	2.13 × 10^−2^	1.60 × 10^−8^
Q44R to EphA4	1.23 × 10^6^	3.71 × 10^−2^	3.02 × 10^−8^
R45K to EphA4	1.03 × 10^6^	5.48 × 10^−2^	5.30 × 10^−8^
Nb60 to EphA6	2.77 × 10^5^	2.30 × 10^−1^	8.29 × 10^−7^
D61R to EphA6	4.17 × 10^5^	2.04 × 10^−1^	4.90 × 10^−7^
D61K to EphA6	4.47 × 10^5^	2.18 × 10^−1^	4.87 × 10^−7^
Q44R to EphA6	4.84 × 10^5^	1.58 × 10^−1^	3.27 × 10^−7^
R45K to EphA6	2.78 × 10^5^	3.57 × 10^−1^	1.29 × 10^−6^

Association and dissociation kinetics were measured by biolayer interferometry. The association rate constant (k_a_) is reported in M^−1^s^−1^ and describes the rate of complex formation between the immobilized receptor LBD and the nanobody analyte. The dissociation rate constant (k_dis_) is reported in s^−1^ and describes the rate of complex dissociation. The apparent equilibrium dissociation constant (K_D_) is reported in M and was calculated as k_dis_/k_a_. Lower KD values indicate stronger apparent binding affinity under the tested BLI conditions.

These results identify D61R and D61K as first-generation Nb60 variants with improved apparent EphA4 binding and enhanced apparent EphA4-over-EphA6 selectivity under matched BLI conditions. More broadly, they support the idea that the noncanonical Nb60 interface is experimentally tunable and that sequence differences among Eph receptor family members can be used to guide structure-based optimization of Nb specificity.

## Discussion

### Structural basis for Nb-mediated competitive inhibition of ephrin binding to EphA4

EphA4 is unique among Eph receptors in its ability to bind both ephrin-A and ephrin-B ligands with high affinity, and aberrant activation of EphA4-ephrin signaling has been implicated in impaired axonal regeneration in ALS (54, 55, 56, 57). In ALS patients and disease models, EphA4 expression is upregulated, and ephrin-A5 has been shown to potently induce EphA4 clustering and autophosphorylation, leading to sustained activation of the Ephexin1-RhoA-ROCK pathway and subsequent growth cone collapse and inhibition of axonal regeneration (30, 51, 58, 59). In parallel, ephrin-B2 and ephrin-B3 are markedly upregulated in activated astrocytes within ALS-affected spinal cord regions, where ephrin-B2 not only promotes forward signaling through EphA4 to suppress axonal growth but may also trigger reverse signaling in astrocytes, potentially exacerbating neuroinflammatory responses and motor neuron degeneration (58, 60). These observations position ligand engagement as a rational therapeutic intervention point for EphA4 inhibition.

**Figure 10:**
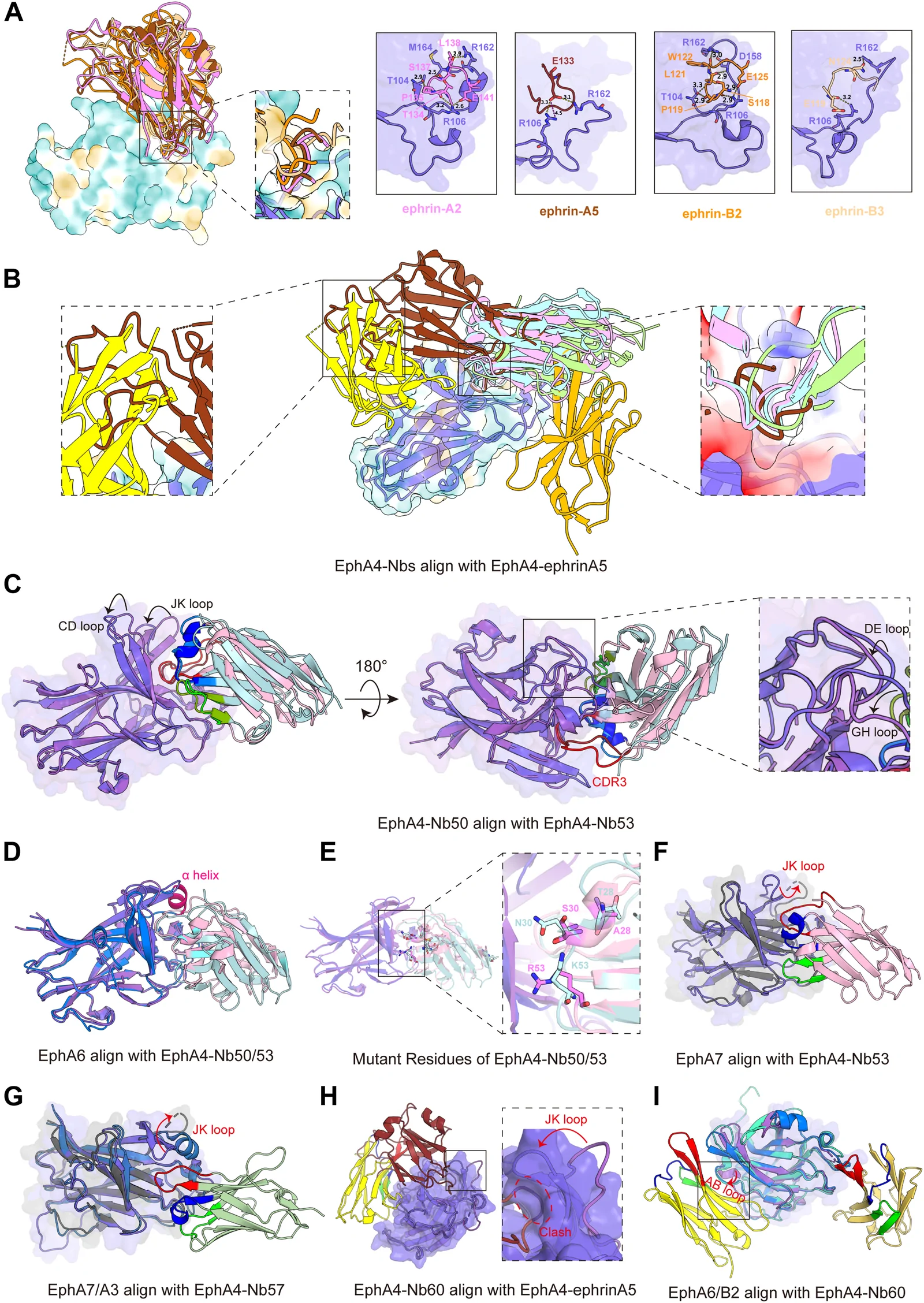
**Structural alignment of EphA4-nanobody complexes with Eph and ephrin family members.***A*, interaction analysis of EphA4 with ephrinA2, ephrinA5, ephrinB2, and ephrinB3. EphA4 is shown in *blue-purple*, ephrinA2 in *violet*, ephrinA5 in *brown* (PDB ID: 4BKA), ephrinB2 in *orange*, and ephrinB3 in *light brown* (PDB ID: 4BKF). *B*, structural alignment of EphA4-nanobody complexes with the EphA4-ephrinA5 complex. Nb50 in *light blue*, Nb53 in *light pink*, Nb57 in *light green*, and Nb60 in two distinct binding modes shown in *bright yellow* and *orange*. *C*, alignment of EphA4-Nb50 with EphA4-Nb53. EphA4-Nb50 is shown in *blue-purple*/*light blue* with CDR1, CDR2, and CDR3 colored *blue*, *green*, and *red*, respectively. EphA4-Nb53 is shown in *purple*/*light pink* with CDR1, CDR2, and CDR3 colored *cyan*, *dark green*, and *dark red*, respectively. *D*, alignment of EphA6 with the EphA4-Nb50 and EphA4-Nb53 complexes. EphA6 (an AlphaFold-predicted model of EphA6) is shown in *dark blue*, with α-helical regions markedly different from EphA4 highlighted in magenta. *E*, sequence differences between Nb50 and Nb53 mapped onto the EphA4-nanobody complexes. Differential residues are shown as *sticks*. *F*, alignment of EphA4-Nb53 with EphA7. EphA7 (PDB ID: 3NRU) is shown in *black*. *G*, alignment of EphA7 and EphA3 with the EphA4-Nb57 complex. EphA3 (PDB ID: 4L0P) is shown in *sky blue*. *H*, alignment of the EphA4-Nb60 complex with the EphA4-ephrinA5 complex. The EphA4-Nb60 binding model 1 is shown in *light purple*/*yellow* with CDR1, CDR2, and CDR3 colored *blue*, *green*, and *red*, respectively. *I*, alignment of EphA6 and EphB2 with the EphA4-Nb60 complex. EphA4 is shown in *purple*, and EphB2 (PDB ID: 3ETP) is shown in *bright green*. CDR, complementarity-determining region; EphA, erythropoietin-producing hepatocellular carcinoma A; Nb, nanobody.

Structural comparison with previously reported EphA4-ephrin complexes (*e.g.* PDB IDs: 2WO2, 2WO3, 4BKA, and 4BKF) reveals that ephrin recognition relies on a highly conserved hydrophobic pocket within the LBD (51, 52, 61), stabilized by key positively charged residues such as Arg106 and Arg162 (Fig. 10*A*). This conserved architecture provides a structural template that can be readily exploited by competitive inhibitors.

Our structural analyses demonstrate that Nb50, Nb53, and Nb57 adopt binding modes that closely mirror ephrin engagement. These Nbs insert their flexible CDR3 loops into the canonical ephrin-binding pocket, directly contacting residues critical for ligand recognition and forming extensive, complementary interaction interfaces. Such ligand-mimetic binding provides a structural rationale for their competitive inhibition of ephrin binding and potential suppression of EphA4 activation (Fig. 10*B*).

Notably, while most EphA4-targeting Nbs conform to this canonical competitive inhibition strategy, not all inhibitory binding modes are restricted to direct pocket occupation. As discussed below, Nb60 exemplifies an alternative mechanism that does not involve direct occupation of the ephrin-binding pocket, suggesting a distinct mode of receptor modulation (Fig. 10*B*).

### Structural determinants of Nb selectivity toward EphA4

Although the EphA4 LBD adopts a highly conserved β-sandwich fold across all Nb-bound structures (52, 62), EphA4-targeting Nbs display markedly different selectivity profiles toward other Eph family members (Fig. S4). This apparent discrepancy underscores that receptor selectivity is not dictated by global fold recognition, but instead arises from subtle differences in epitope usage, binding geometry, and conformational adaptability (Fig. S5).

A particularly illustrative example is provided by Nb50 and Nb53, which differ by only seven amino acids and share an identical CDR3 sequence, yet exhibit strikingly distinct selectivity to EphA6 (Fig. S7) (50). Structural analyses indicate that these sequence differences modulate the balance between flexibility and rigidity within the Nb paratope (Fig. 10*C*). Nb53 appears to adopt a more rigid and hydrophobic binding configuration that promotes deeper engagement with the EphA4 binding cavity, supported by additional hydrogen-bonding interactions mediated by CDR1 residue Arg53 (Fig. 10*E*). This tighter and more geometrically constrained interaction favors EphA4 recognition but reduces tolerance toward closely related receptors such as EphA6, whose LBD is predicted to impose steric constraints incompatible with the broader Nb53 CDR3 conformation (Fig. 10*D*).

Residual cross-reactivity of Nb53 toward EphA7 further highlights the role of receptor-side conformational plasticity in shaping selectivity. Although EphA7 lacks several EphA4 residues that stabilize Nb53 binding, increased flexibility and outward displacement of the JK loop may partially compensate for these structural differences, allowing suboptimal but detectable engagement (Fig. 10*F*). These observations suggest that Nb selectivity emerges from an interplay between paratope geometry and receptor loop adaptability, rather than from a single dominant determinant.

In contrast, Nb57 employs a more compact binding strategy characterized by closely clustered CDR loops and a dominant contribution from CDR3. This architecture enables effective insertion into the conserved β-sheet cavity of EphA4 while remaining permissive to structural variations among other Eph receptors. As a consequence, Nb57 accommodates additional features such as the α-helical insertion present in EphA3 or the more open JK loop of EphA7, resulting in broader but weaker cross-reactivity at the expense of strict receptor selectivity (Fig. 10*G*) (50).

Together, these comparisons reveal that Nb selectivity toward EphA4 is governed not by affinity alone, but by how paratope geometry, loop flexibility, and epitope conservation collectively shape compatibility with related receptors. This framework provides a structural rationale for tuning Nb specificity through targeted modulation of binding topology rather than simple optimization of binding strength.

### Noncanonical Nb60 binding provides a structural model for allosteric modulation of EphA4

In contrast to Nbs that inhibit EphA4 through direct competition with ephrin ligands, Nb60 engages a spatially separated, noncanonical surface on the EphA4 LBD, thereby avoiding direct ligand mimicry (Fig. 10*B*). This binding mode is associated with an inward displacement of the JK loop and a contracted ligand-binding pocket conformation (Fig. 10*H*), providing a structural model by which Nb60 could restrict ephrin access allosterically without directly occupying the canonical ligand-recognition site.

The EphA4-Nb60 crystal structure further revealed a multivalent arrangement in which Nb60 contacts two EphA4 LBD molecules. However, this observation should be interpreted cautiously. The secondary Nb60-EphA4 interface is small, and our solution-state DLS analysis did not reveal evidence of stable high-molecular-weight EphA4-Nb60 oligomers under the tested conditions. Taken together, although the crystal structure defines a 4:2 EphA4-Nb60 assembly, the hydrodynamic radius measured by DLS is more consistent with a compact low-order EphA4-Nb60 complex than with a stable higher-order oligomer under the tested conditions. This difference suggests that the secondary Nb60-EphA4 contact is weak or context-dependent and may be preferentially stabilized in the crystal environment.

Notably, Nb60 relies extensively on framework-mediated interactions and an extended binding interface, distinguishing it from Nbs that predominantly utilize CDR3-driven pocket engagement. This interaction strategy enables recognition of a noncanonical epitope on EphA4 and provides a structural basis for targeting EphA4 outside the conserved ephrin-binding pocket. Such framework-dominated binding expands the accessible epitope space on extracellular receptors and highlights a key advantage of the Nb scaffold for targeting regulatory surfaces that are poorly accessible to conventional antibodies or small molecules (Fig. 10*I*).

More broadly, Nb60 exemplifies an alternative paradigm for extracellular receptor modulation, in which noncanonical epitope recognition and multivalent engagement may provide allosteric control over receptor function. For EphA4, whose canonical ligand-binding pocket is highly conserved across paralogs, this strategy offers a potential route to selective allosteric modulation, including potential inhibition of ligand-dependent activation through mechanisms that do not require direct occupation of the ephrin-binding pocket. At the same time, the multivalent geometry observed for Nb60 raises the possibility that its engagement could alter EphA4 receptor organization and signaling. Depending on the receptor context, such multivalent engagement could lead to either inhibitory or agonistic effects, representing a potential limitation that should be considered when interpreting the functional implications of Nb60 binding. Together, these findings extend the mechanistic repertoire of Nb-mediated EphA4 modulation and provide a conceptual framework for the rational design of allosteric EphA4-targeted modulators in ALS and related neurodegenerative contexts. This framework also prompted us to further examine how epitope selection and binding mode contribute to Nb selectivity among Eph receptor family members.

### Epitope-dependent selectivity of EphA4-targeting Nbs

Selective targeting of individual Eph receptors remains a central challenge due to the high degree of sequence and structural conservation within the ephrin-binding interface. Our structural analyses reveal that Nb selectivity is strongly influenced by epitope location and binding mode rather than overall binding affinity alone. Nbs that engage the canonical ligand-binding pocket, such as Nb50, Nb53, and Nb57, primarily recognize conserved structural features shared among Eph family members, thereby tending to exhibit limited receptor selectivity.

In contrast, Nbs that target noncanonical surfaces display enhanced potential for selectivity by exploiting regions of greater sequence divergence and structural plasticity. The unique epitope recognized by Nb60 lies outside the conserved ephrin-binding pocket and involves residues that are less conserved across Eph receptors. This binding mode provides a structural rationale for differential Eph receptor recognition and for specificity engineering of Nb60, underscoring the importance of epitope choice in achieving receptor discrimination within closely related protein families. Consistent with this concept, our BLI analysis showed that the structure-guided Nb60 variants D61R and D61K increased apparent EphA4-over-EphA6 selectivity under matched assay conditions.

Beyond epitope location, the extent of framework participation further contributes to selectivity. Unlike pocket-binding Nbs that rely predominantly on CDR3-mediated interactions, Nb60 employs an extended interaction interface involving framework regions. Such framework-dominated binding may impose stricter geometric and chemical constraints on receptor recognition, thereby providing a basis for tuning promiscuity toward closely related Eph paralogs. Together, these observations suggest that selective EphA4 modulation may be achieved by combining noncanonical epitope targeting with expanded binding interfaces, rather than by optimizing affinity for conserved ligand-binding sites. More broadly, these findings indicate that targeting peripheral regulatory surfaces may represent a generalizable strategy for achieving subtype-selective receptor modulation within conserved receptor families.

### Limitations and outlook

The present study focuses on the EphA4 LBD as a tractable system for dissecting Nb-receptor interactions at high resolution. While this reductionist approach allows us to resolve binding modes and potential inhibitory mechanisms with clarity, it does not fully capture the architectural and regulatory complexity of the full-length receptor in its membrane-embedded and signaling-competent state. In particular, interdomain contacts, membrane-proximal constraints, local receptor density, and receptor clustering may influence how Nb binding is coupled to receptor activation and signaling *in vivo*.

Similarly, the multivalent EphA4-Nb60 assemblies characterized here are defined primarily through crystallographic and *in vitro* biochemical analyses. These observations provide a compelling structural framework for potential allosteric modulation, including possible restriction of ephrin access, but the precise organization, dynamics, and functional consequences of such assemblies in a cellular environment remain open questions. In particular, although Nb60-mediated multivalent engagement could suppress ligand-dependent EphA4 activation by limiting ephrin accessibility or altering the ligand-binding pocket conformation, it could also, in principle, promote receptor proximity or clustering and lead to agonistic or partial agonistic signaling in the context of full-length EphA4 at the cell surface, where EphA4 signaling is coupled to receptor clustering and higher-order assembly (63, 64). Future studies using full-length EphA4 in signaling-competent systems will help clarify the cellular relevance of these alternative outcomes.

Despite these limitations, the mechanistic insights gained from this work extend beyond the specific system examined. By revealing how epitope selection, binding topology, and multivalency shape Nb-mediated modulation of EphA4, this study offers a foundation for the rational exploration of noncanonical and allosteric modulation strategies. Such principles may extend to other receptor tyrosine kinases and extracellular signaling proteins implicated in ALS and related neurodegenerative conditions. Taken together, these considerations suggest that noncanonical and allosteric Nb binding represents a promising direction for selectively modulating EphA4 and related receptors, while highlighting receptor context as an important determinant of their functional activity.

## Conclusion

In summary, our work provides a structural explanation for how Nbs modulate EphA4 through distinct binding strategies. The identification of a noncanonical, allosteric binding mode alongside classical ligand-competitive binding underscores the versatility of the Nb scaffold. Moreover, structure-guided Nb60 mutagenesis demonstrates that this noncanonical interface can be tuned to improve apparent EphA4-over-EphA6 selectivity. At the same time, the potential of Nb60-like Nbs to promote multivalent receptor engagement highlights the need to determine whether such molecules act as antagonists, partial agonists, or context-dependent modulators in full-length, signaling-competent cellular systems. These findings offer a basis for exploiting alternative epitopes and binding topologies to achieve selective modulation of Eph receptors, with potential relevance for the design of EphA4-targeted approaches in ALS.

## Experimental procedures

### Protein expression and purification

The amino acid sequences of EphA4 LBD-specific Nbs Nb22, Nb31, Nb50, Nb53, Nb57, and Nb60 were identical to those previously reported by Schoonaert *et al.* (50). Codon optimization of the genes encoding EphA4 and the Nbs was performed by Genewiz, Generay and Genecreate. The EphA4 LBD corresponding to residues 30 to 203 was cloned into the pET28a-SUMO and pMAL-c2X expression vectors. An N-terminal His tag followed by a SUMO tag was fused to each Nb to facilitate expression and purification. EphA4 LBD and Nbs were expressed in *E. coli* BL21 (DE3).

Single bacterial colonies were inoculated into 6 ml of LB medium and cultured at 37 °C for 8 to 12 h. The starter cultures were then diluted 1:500 into 1 L of fresh medium and grown at 37 °C until the optical density at 600 nm reached approximately 0.6. Protein expression was induced with 0.2 mM IPTG, followed by incubation at 18 °C for 21 h. Bacteria were harvested by centrifugation at 5000 rpm for 20 min at 4 °C. Bacteria were resuspended in NiA buffer (20 mM imidazole, 5% glycerol, 150 mM NaCl, 100 mM Tris-HCl, pH 7.5) and lysed using a high-pressure homogenizer at 750 bar and 4 °C until complete cell lysis was achieved. Insoluble material was removed by centrifugation at 17,500 rpm for 50 min at 4 °C, and the supernatant was collected for affinity purification.

Ni-NTA Beads 6FF (Smart-Lifesciences, China) were equilibrated with 10 column volumes (CV) NiA buffer prior to use. The clarified supernatant was incubated with the Ni-NTA resin at 4 °C for approximately 1 h. The flow-through fraction was collected for subsequent analysis. The resin was washed sequentially with 6 CV NiA buffer, 5 CV NiC buffer (100 mM Tris-HCl, pH 7.5, 20 mM imidazole, 5% glycerol, 2 M NaCl), and an additional 10 CV NiA buffer. Affinity tags were removed by overnight proteolytic cleavage at 4 °C using ULP1 or TEV protease, depending on the fusion tag used. Cleaved proteins were eluted with 5 CV NiA buffer.

To further improve purity, the proteins were subjected to anion-exchange chromatography using a HiTrap Q column to remove contaminants derived from Ni-affinity purification. Final purification was performed by size-exclusion chromatography on a Superdex 200 Increase 10/300 GL column preequilibrated with buffer containing 20 mM HEPES and 150 mM NaCl (pH 8.0). The molecular weight and homogeneity of EphA4-Nb complexes were assessed by SDS-PAGE.

### MALDI-TOF

MALDI-TOF was used to determine the accurate molecular masses of Nbs and EphA4 LBD. Protein samples were buffer-exchanged into 10 mM ammonium acetate using a Superdex 200 Increase 5/150 GL column (Cytiva). Protein samples were then subjected to analysis using an UltrafleXtreme MALDI-TOF/TOF mass spectrometer (Bruker). Bovine serum albumin was used as a calibration standard for mass accuracy. The mixture of sample and matrix (10 mg/ml 2,5-DHB in 70% ACN and 1% TFA) was dropped onto the sample supports. All spectra were obtained in positive linear mode, and mass spectrometric data analysis was performed using the Bruker FlexAnalysis Software (version 3.3).

### Crystallization and data collection

To obtain high-quality EphA4 LBD-Nb complexes, EphA4 LBD and Nbs were mixed at a molar ratio of 1:1.5 and incubated at 4 °C for 1 h. Excess Nb was removed by size-exclusion chromatography using a Superdex 200 Increase 10/300 GL column. Fractions containing EphA4-Nb complexes with optimal purity and stoichiometry were pooled and concentrated to >50 mg/ml using 10 kDa molecular weight cutoff centrifugal concentrators (Millipore, 500 μl format). Prior to crystallization, samples were clarified by centrifugation at 12,000 rpm for 10 min to remove precipitates, air bubbles, and insoluble impurities. Crystallization trials were performed using the sitting-drop vapor diffusion method with a Gryphon GP-28 robotic system (ARI). Each sitting drop consisted of 0.2 μl of protein solution mixed with 0.2 μl of reservoir solution.

Crystals of the EphA4-Nb57 complex were obtained in a reservoir solution containing 8% (v/v) Tacsimate (pH 4.0) and 20% (w/v) PEG 3350. Crystals of the EphA4-Nb50 complex were grown in 0.1 M citric acid (pH 3.5) supplemented with 25% (w/v) PEG 3350. The EphA4-Nb53 complex crystallized in 0.1 M citric acid (pH 4.0) with 20% PEG 6000 and was further optimized using the hanging-drop vapor diffusion method in 0.1 M citric acid (pH 3.9) and 20% PEG 6000. Crystals of the EphA4-Nb60 complex were obtained in conditions containing 2 M ammonium sulfate and 5% (v/v) 2-propanol.

### Structure determination and X-ray diffraction data collection

Crystals of EphA4 LBD-Nb complexes were cryoprotected by supplementing the reservoir solution with 10% (v/v) glycerol prior to flash-cooling in liquid nitrogen. X-ray diffraction data for the EphA4-Nb50 complex were collected at the BL10U2 beamline of the Shanghai Synchrotron Radiation Facility (SSRF), Chinese Academy of Sciences, with diffraction resolutions ranging from 1.51 to 1.57 Å (65). Diffraction data for the EphA4-Nb57 complex were collected at the BL02U1 macromolecular crystallography beamline of SSRF, yielding resolutions between 1.58 and 2.21 Å (66). Diffraction data for the EphA4-Nb53 complex were obtained at the BL17U1 protein complex crystallography beamline of SSRF (67). Data for the EphA4-Nb60 complex were collected at beamline BL32XU of SPring-8 (Super Photon Ring-8 GeV, Japan). For datasets collected at SSRF, diffraction images from the highest-quality crystals were processed using Automatic Aquarium (68). Data collected at SPring-8 were processed using the CCP4 software suite (version 9.0), including generation of free R flags with the freerflag program (69).

Initial phases for all EphA4-Nb complexes were obtained by molecular replacement using Phaser implemented in the CCP4 crystallography suite. The crystal structure of EphA4 LBD (PDB accession code: 4W4Z (70)) and AlphaFold3-predicted Nb models were used as search templates (71). Model building and manual inspection were performed using COOT (version 0.9.8.1) (72), followed by iterative refinement with PHENIX (version 1.20.1) (73), including coordinate refinement and solvent placement, to achieve consistency with the experimental electron density and theoretical amino acid sequences. Interface areas and PISA-predicted Δ^i^G values were calculated using PDB-PISA. Structural figures were generated using PyMOL (version 3.1.6.1) (74) and ChimeraX (version 1.10) (75).

### DLS

DLS was used to measure the hydrodynamic radius distributions of EphA4 LBD, Nbs, and EphA4 LBD-Nb complexes. Protein samples were prepared at a concentration of 1 mg/ml, and 20 μl of each sample was loaded into Nano-ZS90 Zetasizer (Malvern). Measurements were performed at 25 °C with a fixed scattering angle of 90°. The resulting size distributions were displayed as mass percentage (%) as a function of particle radius (nm).

### CD

CD was performed to analyze the secondary structure content of EphA4 LBD, Nbs, and their complexes. Protein samples were buffer-exchanged by dialysis into ddH_2_O containing 5 mM phosphate. Aliquots of 100 to 200 μl protein solutions at a concentration of 0.5 mg/ml were loaded into quartz cuvettes and measured using a Chirascan V100 CD spectrometer (Applied Photophysics). CD spectra were recorded at 25 °C, over a wavelength range of 180 to 260 nm, with a bandwidth of 1 nm and an integration time of 1 s per data point. Spectra of the corresponding buffer (5 mM sodium phosphate) were subtracted as background. Secondary structure content was estimated using the CDNN software package (version 2.1).

### Nanotemper differential scanning fluorimetry (NanoDSF)

NanoDSF was used to assess the thermal stability of protein samples. Proteins were prepared at a concentration of 1 mg/ml, and 10 μl of each sample was loaded into standard capillaries (Capillaries Cat: PR-C002). Measurements were performed using a Prometheus NT.48 NanoDSF instrument (NanoTemper Technologies, Germany). Thermal unfolding was monitored by intrinsic fluorescence of tryptophan (Trp) and tyrosine (Tyr) residues during heating from 20 °C to 95 °C at a ramp rate of 1 °C/min. Fluorescence emission was recorded at 330 nm and 350 nm. Data were analyzed using PR.ThermControl and PR.StabilityAnalysis software.

### ITC

ITC was performed to determine the thermodynamic parameters of interactions between EphA4 LBD and Nbs using a MicroCal PEAQ-ITC instrument (Malvern Panalytical). For each experiment, 200 μl of EphA4 LBD at a concentration of 20 μM was loaded into the sample cell, while 75 μl of Nb solution at 150 μM was loaded into the syringe. Titrations were conducted at 25 °C by injecting 2 μl aliquots of Nb into the EphA4 LBD solution with an interval of 120 s between injections to allow complete return to baseline. A total of 19 injections were performed for each titration. The binding stoichiometry (N), enthalpy change (ΔH), entropy change (ΔS), and dissociation constant (KD) were obtained by fitting the data to a one-site binding model using the MicroCal PEAQ-ITC Analysis Software.

### BLI

BLI was used to quantify the binding kinetics of Nbs toward EphA4 and EphA6 LBDs. His-tagged EphA4 or EphA6 LBD was immobilized on Ni-NTA biosensors in 1 ✕ PBST (consisting of phosphate-buffered saline supplemented with 0.05% Tween-20) running buffer. After receptor loading, sensors were equilibrated in running buffer to establish a stable baseline and then dipped into wells containing serially diluted Nb analytes. A seven-point concentration series was used, with the highest analyte concentration typically set at 500 nM or 2000 nM depending on the interaction tested. Association was monitored in Nb-containing wells, followed by dissociation in running buffer. Sensorgrams were processed by reference subtraction and baseline alignment, and the association and dissociation phases were globally fitted using a 1:1 binding model. The association rate constant k_a_ was reported in M^−1^s^−1^, the dissociation rate constant k_dis_ was reported in s^−1^, and the apparent equilibrium dissociation constant K_D_ was calculated as k_dis_/k_a_ and reported in M. Because receptor LBDs were immobilized on Ni-NTA biosensors, KD values are reported as apparent affinities under the tested BLI conditions. EphA4-over-EphA6 selectivity was calculated as KD(EphA6)/KD(EphA4).

## Data availability

Atomic coordinates and structure factors have been deposited in the Protein Data Bank under accession codes 23UD, 23UE, 23UF, and 23UG. Other datasets generated during and/or analyzed during the current study are available from the corresponding author on reasonable request.

## Supporting information

This article contains supporting information (19, 50).

## Conflict of interest

The authors declare that they have no conflicts of interest with the contents of this article.
