# Supplementary material for: Distinct competitive and allosteric binding modes of nanobodies targeting EphA4
Source: J Biol Chem. 2026 Aug 5;302(9):113394. doi: 10.1016/j.jbc.2026.113394 (PMC13560340; doi:10.1016/j.jbc.2026.113394)
Supplement: Supplementary file 1 [file mmc1.docx]

**Distinct competitive and allosteric binding modes of nanobodies targeting EphA4**

He Jiang^1^, Lulu Ding^1^, Chenlu Yang^1^, Guankai Guo^1^, Chao Zhong^1^, Zhiqiang Ma^1^, Haoran Zhu^1^, Jiahua Zhang^1^, Yang Luo^1^, Zhen Liu^1^, Rui Liu^#1,2^, Yu Ding^#1,2^

**Supplementary data**

Table S1 The amino acid sequences of EphA4 LBD, EphA6 LBD and nanobodies.

| Protein | Amino acid sequence | Molecular weight |
| --- | --- | --- |
| Nb22 | QVQLQESGGGPVRAGESLRLSCTASGRAFSTYTMGWFRQAPGKERELVAVITPGGNTNYADSVKGRFTISRDDAKNTVYLQMNSLKPVDTAVYYCHVVLAVVGTGNEYDVWGQGTQVTVSS | 13.03 kDa |
| Nb31 | QVQLQESGGGLVQAGGSLRLSCAASGFASDAYAIGWFRQAPGTKREMIAAVTKAGATNYANPVKGRFTISRDDAKNMVYLQMNNLKPEDSAVYYCNAISPLWDEYDFWGPGTQVTVSS | 12.71 kDa |
| Nb50 | QVQLQESGGGLVQAGGSLRLSCAASGRTFNSYAEAWFRQAPGKEREMIAAVTKAGTTNYANPVKGRFTISRDDAKNMVYLQMNNLKPEDSAVYYCNAISPLWDEYDFWGPGTQVTVSS | 12.91 kDa |
| Nb53 | QVQLQESGGGVVQAGGSLRLSCAASGRAFSSYAMGWFRQAPGTKREMIAAVTRAGTTNYANPVKGRFTISRDDAKNMVYLQMNNLKPEDSAVYYCNAISPLWDEYDFWGPGTQVTVSS | 12.83 kDa |
| Nb57 | QVQLQESGGGLVQPGGSLRLSCAASGFTFSSYYMYWVRQAPGKGLEWVSTINTGGGSTNYADSVKGRFIISRDNAKNTVYLQMNSLKPEDTAVYYCNAFAAGWGPDYWGQGTQVTVSS | 12.73 kDa |
| Nb60 | QVQLQESGGGLVQPGGSLRLSCAASGFTFSRYWIYWVRQAPGKQRELVAAITRNGVTNYADSVKGRFTISTDDNKNTVYLQMNSLKSEDTAVYYCNAWGFLLGDIEDYWGQGTQVTVSS | 13.62 kDa |
| EphA4 LBD | EVTLLDSRSVQGELGWIASPLEGGWEEVSIMDEKNTPIRTYQVCNVMEPSQNNWLRTDWITREGAQRVYIEIKFTLRDCNSLPGVMGTCKETFNLYYYESDNDKERFIRENQFVKIDTIAADESFTQVDIGDRIMKLNTEIRDVGPLSKKGFYLAFQDVGACIALVSVRVFYKKA | 20.60 kDa |
| EphA6 LBD | QVVLLDTTTVLGELGWKTYPLNGWDAITEMDEHNRPIHTYQVCNVMEPNQNNWLRTNWISRDAAQKIYVEMKFTLRDCNSIPWVLGTCKETFNLFYMESDESHGIKFKPNQYTKIDTIAADESFTQMDLGDRILKLNTEIREVGPIERKGFYLAFQDIGACIALVSVRVFYKK | 20.12 kDa |

The amino acid sequences of Nb22, Nb31, Nb50, Nb53, Nb57, and Nb60 were based on the sequences previously reported by Schoonaert et al. (50).

Table S2 The detailed information of the PISA analysis on the interaction between nanobodies and EphA4 LBD.

| Interaction | Protein | interface area, Å^2^ | Number of atoms interface | Number of atoms surface | Number of residues interface | Number of residues surface | Solvent-accessible area, Å interface |
| --- | --- | --- | --- | --- | --- | --- | --- |
| EphA4-Nb50 | EphA4 | 1072.0 | 114 (8.7%) | 779 (59.2%) | 32 (19.3%) | 160 (96.4%) | 1089.5 (12.0%) |
|  | Nb50 |  | 121 (13.7%) | 535 (60.6%) | 28 (23.9%) | 110 (94.0%) | 1054.6 (16.9%) |
| EphA4-Nb53 | EphA4 | 1131.9 | 118 (8.5%) | 805 (58.2%) | 36 (21.1%) | 160 (93.6%) | 1112.3 (11.8%) |
|  | Nb53 |  | 127 (14.3%) | 530 (59.5%) | 30 (25.6%) | 106 (90.6%) | 1151.5 (17.8%) |
| EphA4-Nb57 | EphA4 | 807.6 | 78 (5.6%) | 823 (59.3%) | 26 (14.9%) | 163 (93.7%) | 792.6 (8.1%) |
|  | Nb57 |  | 86 (10.1%) | 472 (55.2%) | 18 (15.9%) | 100 (88.5%) | 822.7 (13.9%) |
| EphA4-Nb60  Mode 1 | EphA4 | 860.0 | 82 (5.9%) | 853 (60.9%) | 26 (15.0%) | 166 (96.0%) | 873.1 (9.2%) |
|  | Nb60 |  | 98 (11.3%) | 504 (57.9%) | 26 (23.0%) | 104 (92.0%) | 846.9 (14.1%) |
| EphA4-Nb60  Mode 2 | EphA4 | 459.4 | 40 (3.1%) | 743 (56.8%) | 10 (6.1%) | 154 (94.5%) | 485.0 (5.4%) |
|  | Nb60 |  | 55 (6.3%) | 504 (57.9%) | 16 (14.2%) | 104 (92.0%) | 433.9 (7.2%) |

The Δ^i^G values were calculated by PDB-PISA from crystallographic interfaces and represent computationally predicted interface energy estimates, not experimentally measured binding free energies.

Table S3 BLI binding affinity data for six nanobodies to EphA4 LBD.

| **Group** | **k_a_ (1/Ms)** | **K_dis_ (1/s)** | **K_D_ (M)** |
| --- | --- | --- | --- |
| Nb22 to EphA4 | 2.46🞨10^5^ | 8.15🞨10^-3^ | 3.32🞨10^-8^ |
| Nb31 to EphA4 | 3.16🞨10^5^ | 8.75🞨10^-2^ | 2.77🞨10^-7^ |
| Nb50 to EphA4 | 9.45🞨10^5^ | 1.18🞨10^-1^ | 1.25🞨10^-7^ |
| Nb53 to EphA4 | 9.36🞨10^5^ | 5.11🞨10^-2^ | 5.47🞨10^-8^ |
| Nb57 to EphA4 | 1.19🞨10^6^ | 1.85🞨10^-2^ | 1.55🞨10^-8^ |
| Nb60 to EphA4 | 7.85🞨10^5^ | 3.18🞨10^-2^ | 4.05🞨10^-8^ |

The association rate constant k_a_ (1/Ms) represents the rate of analyte association with the immobilized receptor, whereas the dissociation rate constant k_dis_ (1/s) represents the rate of complex dissociation. The apparent dissociation constant K_D_ (M) was calculated as k_dis_/k_a_.

Figure S1. Size-exclusion chromatography profiles of nanobodies, EphA4 LBD, and EphA4 LBD-nanobody complexes.

(A-F) Nb22, Nb31, Nb50, Nb53, Nb57, and Nb60, respectively; (G) EphA4 LBD; (H–M) EphA4 LBD complexes with Nb22, Nb31, Nb50, Nb53, Nb57, and Nb60, respectively.

Figure S2. SDS-PAGE analysis of EphA4 LBD, nanobodies, and EphA4 LBD–nanobody complexes.

M, molecular weight marker; lanes 1 and 2, EphA4 LBD purified from two independent preparations.

Figure S3. Molecular weight determination of EphA4 LBD-nanobody complex by MALDI-TOF mass spectrometry.

(A-F) EphA4 LBD complexes with Nb22, Nb31, Nb50, Nb53, Nb57, and Nb60, respectively.

Figure S4. Overview of the EphA4 ligand-binding domain.

The structure was generated from the previously reported EphA4 structure (PDB ID: 2WO1) (19). The N and C termini are labelled. The β-strand are labelled, and the α-helix are shown in cyan.

Figure S5. ESPript 3 sequence alignment of Eph family proteins.

Key EphA4 LBD residues involved in Nb60 recognition in binding mode 1 and binding mode 2 are highlighted in purple and green, respectively. Sequence conservation at these sites between EphA4 and EphA6 is shown, and EphA6 residues that differ from the corresponding EphA4 residues are marked with blue circles.

Figure S6. BLI binding analysis of six nanobodies to EphA4.

(A-F) representative sensorgrams for Nb22, Nb31, Nb50, Nb53, Nb57, and Nb60 binding to EphA4 LBD, respectively.

Figure S7. ESPript 3 sequence alignment of nanobodies.

CDR1, CDR2, CDR3 are labelled in blue, green, and red, respectively. The nanobody sequences used for the alignment were previously reported (50).
